# The reactive vaccination campaign against cholera emergency in camps for internally displaced persons, Borno, Nigeria, 2017: a two-stage cluster survey

**DOI:** 10.1136/bmjgh-2020-002431

**Published:** 2020-06-29

**Authors:** Moise Chi Ngwa, Wondimagegnehu Alemu, Ifeanyi Okudo, Collins Owili, Uzoma Ugochukwu, Peter Clement, Isabelle Devaux, Lorenzo Pezzoli, James Agada Oche, Chikwe Ihekweazu, David A Sack

**Affiliations:** 1 International Health, Johns Hopkins University Bloomberg School of Public Health, Baltimore, Maryland, USA; 2 WHO, Nigeria Country Office, Abuja, Nigeria; 3 WHO Country Office, Maiduguri, Nigeria; 4 WHO, Geneve, Switzerland; 5 Nigeria Centre for Disease Control, Abuja, Nigeria

**Keywords:** cross-sectional survey, cholera, public health, vaccines

## Abstract

**Introduction:**

In 2017, amidst insecurity and displacements posed by Boko Haram armed insurgency, cholera outbreak started in the Muna Garage camp for Internally Displaced Persons (IDPs) in Borno State, Nigeria. In response, the Borno Ministry of Health and partners determined to provide oral cholera vaccine (OCV) to about 1 million people in IDP camps and surrounding communities in six Local Government Areas (LGAs) including Maiduguri, Jere, Konduga, Mafa, Dikwa, and Monguno. As part of Monitoring and Evaluation, we described the coverage achieved, adverse events following immunisation (AEFI), non-vaccination reasons, vaccination decisions as well as campaign information sources.

**Methods:**

We conducted two-stage probability cluster surveys with clusters selected without replacement according to probability-proportionate-to-population-size in the six LGAs targeted by the campaign. Individuals aged ≥1 years were the eligible study population. Data sources were household interviews with vaccine card verification and memory recall, if no card, as well as multiple choice questions with an open-ended option.

**Results:**

Overall, 12 931 respondents participated in the survey. Overall, 90% (95% CI: 88 to 92) of the target population received at least one dose of OCV, range 87% (95% CI: 75 to 94) in Maiduguri to 94% (95% CI: 88 to 97) in Monguno. The weighted two-dose coverage was 73% (95% CI: 68 to 77) with a low of 68% (95% CI: 46 to 86) in Maiduguri to a high of 87% (95% CI: 74 to 95) in Dikwa. The coverage was lower during first round (76%, 95% CI: 71 to 80) than second round (87%, 95% CI: 84 to 89) and ranged from 72% (95% CI: 42 to 89) and 82% (95% CI: 82 to 91) in Maiduguri to 87% (95% CI: 75 to 95) and 94% (95% CI: 88 to 97) in Dikwa for the respective first and second rounds. Also, coverage was higher among females of age 5 to 14 and ≥15 years than males of same age groups. There were mild AEFI with the most common symptoms being fever, headache and diarrhoea occurring up to 48 hours after ingesting the vaccine. The most common actions taken after AEFI symptoms included ‘did nothing’ and ‘self-medicated at home’. The top reason for taking vaccine was to protect from cholera while top reason for non-vaccination was travel/work. The main source of campaign information was a neighbour. An overwhelming majority (96%, 95% CI: 95% to 98%) felt the campaign team treated them with respect. While 43% (95% CI: 36% to 50%) asked no questions, 37% (95% CI: 31% to 44%) felt the team addressed all their concerns.

**Conclusion:**

The campaign achieved high coverage using door-to-door and fixed sites strategies amidst insecurity posed by Boko Haram. Additional studies are needed to improve how to reduce non-vaccination, especially for the first round. While OCV provides protection for a few years, additional actions will be needed to make investments in water, sanitation and hygiene infrastructure.

**Key questions**

**What is already known?**

- In 2017, reactive oral cholera vaccine campaigns were implemented in Borno, Nigeria, with an intent to stop cholera outbreak that began in a camp for internally displaced persons from spreading.

**What are the new findings?**

- Overall, coverage with at least one dose of vaccine was 90% while complete (two-dose) course was 73% and the coverage was lower during the first than second round of campaigns.
- Overall, coverage was higher among females of age 5 to 14 and ≥15 years than males of same age groups.
- Fever was the most common symptom of adverse events following immunisation, and ‘protect from cholera’ and ‘absence during campaign’ were the top reasons for vaccination and non-vaccination.
- Neighbour was the main source of campaign information and an overwhelming majority of target population felt that the campaign team treated them with respect.

**What do the new findings imply?**

- Studies to understand sociocultural and behavioural determinants of vaccine acceptance are urgently needed to guide strategies to improve non-vaccination, especially among mobile populations.

## Introduction

**Figure 1:**
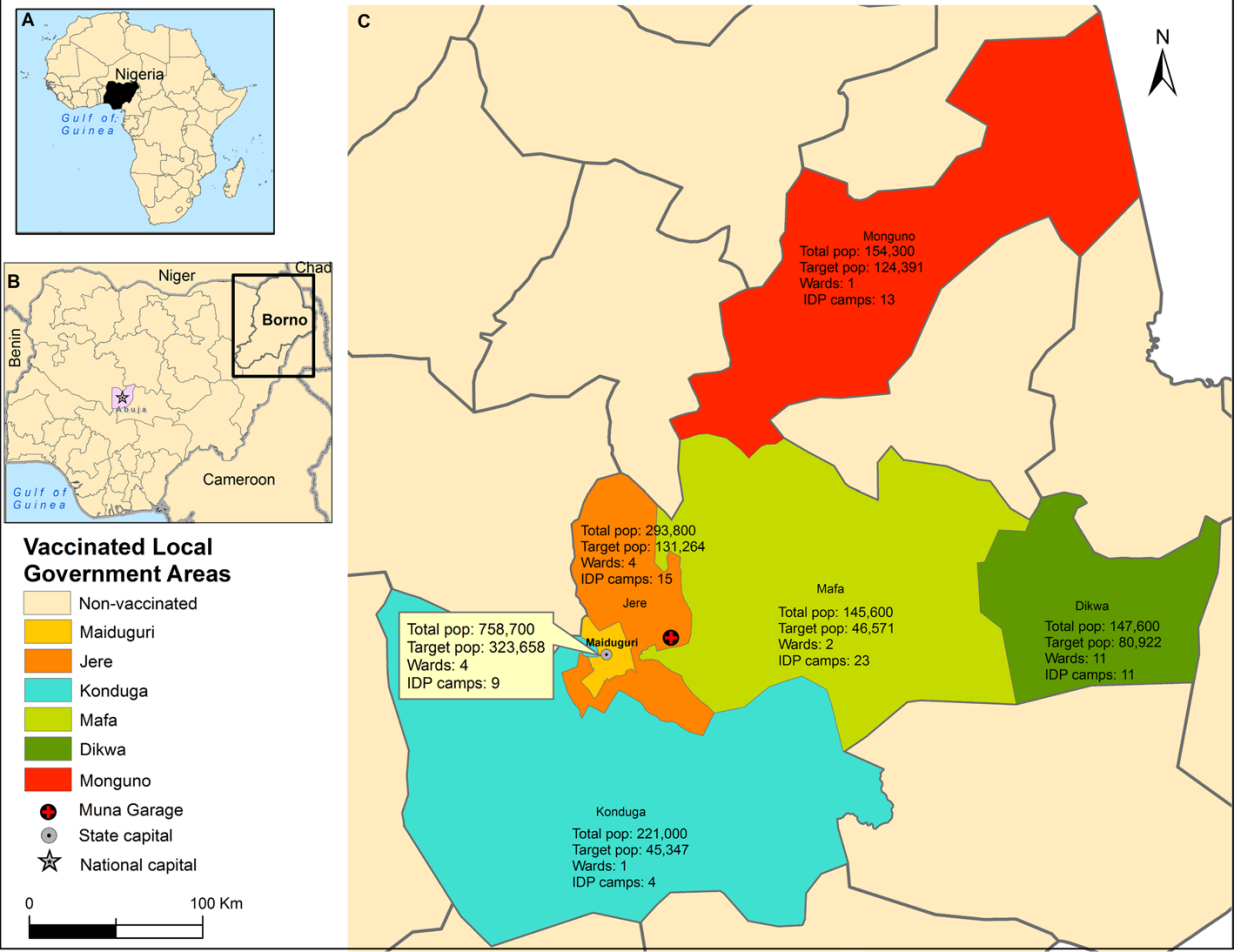
Vaccinated Local Government Areas (LGAs) in Borno, Nigeria - 2017. (A) Nigeria within the continent of Africa. (B) Neighbouring countries to Nigeria, Nigeria capital (Abuja) and Borno State where the first oral cholera vaccine in Nigeria were deployed in 2017. (C) LGA total and target population (pop) of the mass campaigns as well as number of wards and IDPs that were vaccinated. IDP, Internally Displaced Person.

Cholera is an infection of the intestines transmitted in settings with poor water, inadequate sanitation and hygiene. The WHO estimates that cholera causes about 1.3 to 4.0 million cases (21 000 to 143 000 deaths) yearly worldwide,^1^ including Nigeria (figure 1A). When Nigeria reported cholera for the first time in 1970,^2^ an endemic pattern ensued with a burden of 321 421 reported cases (case fatality ratio (CFR) 5.8%) between 1991 and 2019.^2 3^ This burden is greatest in the northeast^4^ where humanitarian crises linked with floods and Boko Haram conflict^5^ forced an estimated 2.6 million people^6^ into camps for Internally Displaced Persons (IDPs) with insufficient food, clean water, proper sanitation and hygiene. These make the northeast of Nigeria including Borno (figure 1B) at high risk of cholera. Very large cholera outbreaks were associated with conflicts in South Sudan, Iraq, Somalia and Yemen^7^ while floods were associated with many outbreaks in sub-Saharan Africa between 1990 and 2010.^8^ In 2010, floods preceded outbreaks in Borno that left 21 111 cases (CFR 5.1%) in its wake^9^ while the 2017 Muna Garage IDP camp outbreak in Jere (figure 1C) caused 5340 cases (CFR 1.14%).^10^ As an emergency response^11^ to the latter outbreak, the Nigeria government organised a reactive oral cholera vaccine (OCV) campaign in Borno as part of integrated measures with an intent to stop the spread of the outbreak. Mass vaccination campaigns were conducted in six Local Government Areas (LGAs) with focus on IDP camps and surrounding villages, targeting about one million people. Although quantitative surveys have documented OCV use in IDP camps,^12–16^ no such surveys have analysed OCV use in IDP camps in Borno in particular and in Nigeria in general.

### Decision to use oral cholera vaccine in Borno

**Figure 2:**
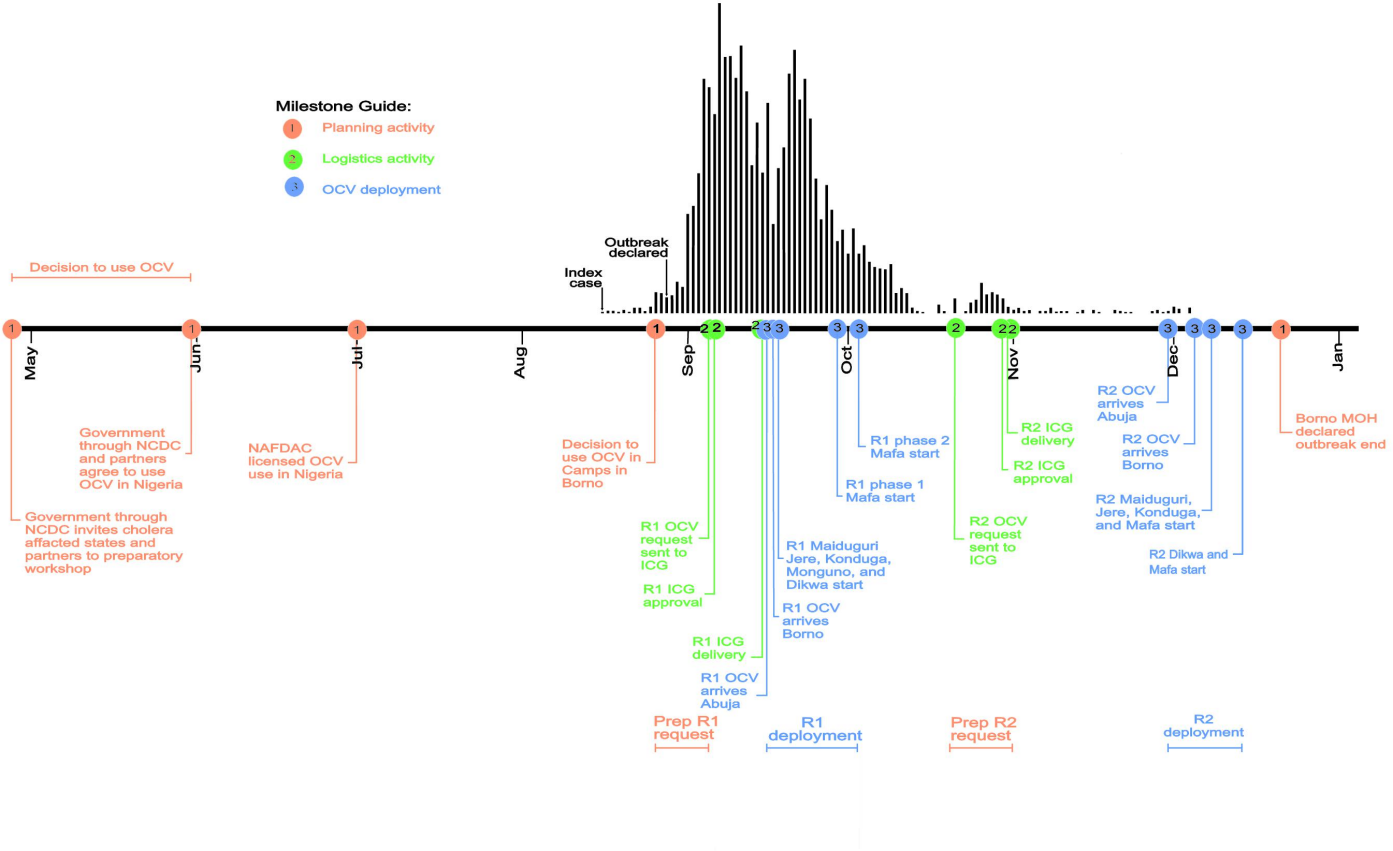
Timeline of major activities of mass oral cholera vaccine campaigns in Borno, Nigeria 2017. (R1) and (R2) indicate rounds 1 and 2 campaigns, respectively. The red (planning activity), green (logistic activity) and blue (OCV deployment) dots on the timeline represents key milestones, while the colours represent the various phases leading up to the mass campaigns in Borno. The epidemic curve of the 2017 Borno outbreak is shown by the vertical bars above the timeline. Note that the decision to use OCV in Nigeria was not linked with the Borno outbreak; the latter only gave opportunity to implement decisions that were taken in May/June 2017. ICG, International Coordinating Group; MOH, Ministry of Health; NAFDAC, National Agency for Food and Drug Administration and Control; NCDC, Nigeria Centre for Disease Control; OCV, oral cholera vaccine.

Between 31^st^ May and 1^st^ June 2017, the Nigeria government through the Nigeria Centre for Disease Control held a cholera preparedness workshop where cholera affected states were invited, including Borno. This workshop, prior to the outbreak, discussed the idea of using OCV in Nigeria and recommended that a license for OCV be obtained to facilitate the use of vaccine in the country; OCV was then licensed by the National Agency for Food and Drug Administration and Control 6 weeks later (figure 2). With the licensing of OCV, the 16^th^ August 2017 outbreak in Borno afforded the opportunity to use OCV for the first time in Nigeria with the first request sent to the International Coordinating Group (ICG)^17^ on 5^th^ September 2017. With ICG’s immediate approval, 915 005 doses of Shanchol (Shantha Biotechnics, Sanofi Company) arrived in Abuja 10 days later and were delivered to Borno within 2 days. As it was not readily apparent if there was sufficient vaccine available for round 2, the second application to ICG had a time lag of 6 weeks from the first. With ICG’s approval of second request 9 days after receiving the application, 896 919 doses of Shanchol arrived in Abuja 1 month later (figure 2).

The National Primary Healthcare Development Agency in collaboration with WHO, United Nations Children's Fund, Médecins Sans Frontières and other implementing partners assisted the Borno Ministry of Health to implement the OCV campaigns in Borno. During two rounds the campaign immunised people 1 year and above (excluding pregnant women) using combined door-to-door and fixed site strategies. The first round of OCV was delivered within 5 days (18^th^ to 22^nd^ September 2017) targeting 855 492 individuals in Maiduguri, Jere, Konduga, Dikwa and Monguno LGAs. The first round of campaign in Mafa was divided into two phases from 29^th^ September to 1^st^ October 2017 and from 3^rd^ to 4^th^ October 2017. Overall, a total of 896 919 doses were administered. The second round was also implemented within 5 days in Maiduguri, Jere and Mafa from 8^th^ to 12^th^ December 2017, while those in Dikwa, Konduga and Monguno took place from 14^th^ to 18^th^ December 2017 (figure 2).

We undertook post-campaign surveys as part of a Monitoring and Evaluation (M&E) activity^18^ to evaluate the conduct of the campaigns. The primary objective was to estimate coverage: *What proportion of the target population* in *IDP camps and surrounding communities received OCV during the campaigns?* Our secondary objectives were to compare coverage and document vaccine decision-making: (1) *What was the likely population coverage in subpopulations stratified by sex and age groups? and (2) What factors guided the decision to take or not take (non-vaccination) the vaccine?*

## Methods

### Study setting

Borno State with its capital Maiduguri is one of the 36 states of Nigeria located in the northeastern region of the Sahel belt bordering Niger, Chad and Cameroon (figure 1B). It is host to 5 860 200 inhabitants living in 27 LGAs in 2016.^19^ Overall, within the LGAs, the population sizes range from 79 700 in Kwaya Kusar to 758 700 people in Maiduguri. The 27 LGAs are further divided into 308 wards of between 10 to 15 wards per LGA. These population denominators were derived from the Population and Housing Census of the Federal Republic of Nigeria, which was conducted in 2006 using convenient areas called census enumeration areas (EAs),^20^ that is, LGA population estimates were derived from the EAs.^21^ Borno has a total of 164 IDPs camps including 59 at high risk of floods;^22^ and thus, the most affected of the Northern states affected by Boko Haram insurgency. We conducted surveys in the settings of wards and IDP camps in the six LGAs that were vaccinated (figure 1C). The OCV campaigns targeted populations ranging from 45 347 in Konduga LGA to 323 658 in Maiduguri LGA and these constituted the estimated study population numbers for this coverage survey (figure 1C).

#### Staff training

To ensure high-quality data, 47 data collectors and six supervisors received a 4-day training on ethical conduct of interviews. Key topics included standard operating procedures, computer-assisted personal interviewing (CAPI), taking global positioning system (GPS) coordinates, taking photos of vaccination cards and establishing vaccine eligibility (all persons 1 year and above, except pregnant women). In particular, they were trained to establish vaccination status from memory if no card is available. Training methods included PowerPoint presentation, role play and pilot field test. Trainees discussed shortcomings and improvements immediately after role play while the trainer gave feedback on errors immediately after the pilot field practices.

### Study design and sample size calculation

A two-stage probability cross-sectional cluster survey was used following the WHO recommendations.^23^ Estimate of an appropriate sample size was geared towards addressing the aforementioned descriptive questions. The expected coverage was 50% (desired precision level of 10%) with a design effect of 2.5 (to account for correlation of responses within clusters) based on a confidence level of 95% (ie, 5% probability of being wrong). Based on these assumptions, the estimated sample size was 900 households (HHs) in each LGA (ie, 300 HHs for each age strata).

### Sampling frames, selecting clusters and households

For the two-stage probability cluster design, the sampling frame consisted of two forms. Form ward consisted of ward and IDP camp population numbers, and was used as cluster sampling frame (primary sampling unit) for the first stage (online supplementary S1 table). In form HH, we enumerated every household within the selected clusters, which was the sampling frame for the second stage (secondary sampling unit). All inhabited houses, tents and huts were visited by the survey team and given HH identification numbers (IDs); unoccupied units (schools, churches, health facilities, houses, tents and huts) were not given IDs. Where more than one HH occupied a unit, it received a single number, but with distinguishing alphabets (eg, 2a, 2b, 2c and so on).

10.1136/bmjgh-2020-002431.supp1 Supplementary data

Cluster and household selection followed WHO reference manual.^23^ In stage one, 30 clusters per LGA were randomly and systematically selected (online supplementary S1 table) without replacement, using probability proportionate to population size. In stage two, a random sample of 30 households per cluster was selected using the =RAND() formula in Microsoft Excel. This selection was done in the central office in Maiduguri and before the data collectors went to the field.

### Patient and public involvement

Patients or the public were not involved in the development or implementation of this study.

### Data collection and entry and respondents

A mixed-mode design, including face-to-face and CAPI using Android mobile phones, was used to administer interviews after consent. A pilot-tested questionnaire (online supplementary S2 table) was used to capture (1) demographic data (sex, age and HH size), (2) coverage (card-verified and memory recall), (3) Adverse Events Following Immunisation (AEFI) (AEFI symptoms, symptoms start time and action taken), (4) reasons for taking/not taking vaccine, (5) campaign information sources (eg, neighbour, village crier, camp) and (6) campaign experience (whether campaign team treated vaccinees with respect and addressed all their question).

10.1136/bmjgh-2020-002431.supp2 Supplementary data

Using phones for data collection allowed the interviewer and supervisor to check the entries for mistakes and correct them before the data was saved. Pictures of OCV cards were taken following WHO reference manual^23^ to ascertain vaccination dates and were re-examined if incorrect dates were recorded or if errors were made in entering them into the questionnaire forms.

The KoBoCollect application, an android-based application (http://www.kobotoolbox.org/), was used for offline data entry in the field into the mobile devices. The data were uploaded daily (or after work was completed from a distant cluster) to the KoBo sever via mobile hotspot at the emergency operations centre in Jere. Data quality checks were done by (1) including skip patterns in the questionnaire form, and also by (2) data collectors and (3) supervisors in the field.

Following Demographic and Health Survey^24^ and Multiple Indicator Cluster Surveys^25^ survey procedures, eligible respondents included both residents and all other persons 1 year and above who slept in the household the previous night. Identification of cluster boundaries and HHs were done with assistance from ‘Bullemas’, village headmen/community gatekeepers in whom people trust. GPS coordinates were recorded, which helped to identify if a household was within the right geographical cluster. When respondents were not at home during first visit, up to two revisits were conducted to obtain the needed data. When residents were not at home after second revisit, information were obtained from the neighbours or ‘Bullemas’. The questionnaire form listed a field to indicate whether information about eligible but absent HH came from a neighbour or ‘Bullema’.

#### Data analyses

The main outcome of this study was vaccination coverage (complete coverage and at least one dose) in the target population. We estimated coverage for all six LGAs combined and for each LGA. Coverage as evidenced by vaccination card (card-verified) and coverage through self-report (memory recall) if no card is available. We note that card-verified plus self-reported coverage is termed crude coverage. Comparative outcomes included coverage between subgroups such as sex (female/male) and age (1 to 4, 5 to 14 and >15 years).

Other outcomes included occurrence, rate and type of AEFI, reasons for vaccination/non-vaccination, information sources of and vaccinee experience with the campaign. The 95% CIs (Clopper-Pearson intervals) for coverage proportions were estimated using the method of Korn and Graubard.^26^ As data were collected from every eligible respondent in every selected household, appropriate syntax and techniques were used to incorporate the cluster ID, household ID and household resident ID in the estimation to account for the multilevel nature of the data, and for correlated responses from respondents nested within households nested within clusters. To compare coverage between subgroups, Rao-Scott χ^2^ technique was used to account for the stratified systematic cluster survey sampling and weights. Data analysis incorporated weighted statistical analysis techniques that accounted for the complex survey design including appropriate statistical adjustments for missing data. Tables were produced for both the descriptive and comparative outcomes and graphs produced to visualise the results.

In keeping with our objective of comparing OCV coverage by gender and age, we used survey-weighted logistic regression to estimate ORs for coverage by sex and age groups (ie, using sex and age groups as predictor variables of the binary outcome coverage in the logistic regression model.) We fit both overall (all six LGAs combined) and LGA-specific versions of these logistic regression models. We report estimated ORs and associated 95% CIs for sex (OR of coverage for males compared with females) that are (1) unadjusted, (2) adjusted for age group and (3) modified by age, meaning the OR for sex is allowed to change depending on age group.

Analysis was carried out using R Statistical software (The R Foundation for Statistical Computing) and ArcGIS V.10.5.1 (Esri, Redlands, California, USA) was used to produce maps.

### Ethical considerations

This survey was approved by the Borno Ministry of Health as part of M&E of the OCV mass campaign. Based on this, the Johns Hopkins University Internal Review Board (IRB) determined that the proposed study activity does not qualify as human subjects’ research, and so does not require IRB. Verbal informed consent was obtained prior to any interviews and after explaining the purposes of the survey. Adults provided their own consent while consent for children was obtained from parent/guardian. To ensure privacy and prevent unauthorised persons from photos of vaccine cards, only authorised persons had access to the list that indicates which photos are associated with which survey respondents.

## Results

### Descriptive statistics

Overall, surveyors visited 5275 HHs between 7^th^ and 21^st^ February 2018 (table 1) and of these 4596 (87.2%) consented to the survey (online supplementary S1 figure A) and 12 931 individuals were recruited yielding 7371 (57.0%) females (table 1). Females were more likely than males to retain and present vaccine cards on request in interviews. The mean household size was 3 (online supplementary S1 figure B) with IQR=3 while the median age was 12 years old (IQR=25). Finally, the number of HHs visited and individuals included varied by LGA (table 1).

10.1136/bmjgh-2020-002431.supp3 Supplementary data

10.1136/bmjgh-2020-002431.supp4 Supplementary data

**Table 1:** Survey indicators by LGA

LGA	Total no of clusters	Date		Total HHs visited, participation rate (%)	Total respondents, per cent female (%)
		From	To		
Jere	30	2/7/2018	2/14/2018	900 (100)	2710 (55.1)
Maiduguri	29	2/14/2018	2/15/2018	865 (80.8)	2121 (58.0)
Konduga	30	2/14/2018	2/18/2018	851 (84.3)	2391 (57.8)
Mafa	30	2/15/2018	2/21/2018	878 (84.7)	2387 (58.6)
Dikwa	30	2/16/2018	2/20/2018	895 (90.5)	1374 (55.7)
Monguno	30	2/18/2018	2/21/2018	883 (89.3)	1938 (57.0)
**Total**	**179**	─	─	**5272** (**87.2**)	**12 931** (**57.0**)

HHs, households; LGA, Local Government Area.

#### Weighted coverage estimates

##### *All LGAs combined:* Maiduguri, Jere, Konduga, Mafa, Dikwa and Monguno

Overall, 90% (95%CI: 88 to 92) of the population targeted by the campaign received at least one dose of OCV. Weighted complete coverage was 73% (95% CI: 68 to 77) among the target population. The campaign achieved lower coverage during first round (76%; 95% CI: 71 to 80) compared with the second round (87%; 95% CI: 84 to 89) of immunisation. Coverage through vaccine card verification was 55% (95% CI: 50 to 59) in the second round; vaccine cards were not issued during first round. Coverage increased by 11% (95% CI: 9 to 13) between the first and second round, and the highest increase was among the 1- to 4-year female group (online supplementary S3 table A).

10.1136/bmjgh-2020-002431.supp5 Supplementary data

**Figure 3:**
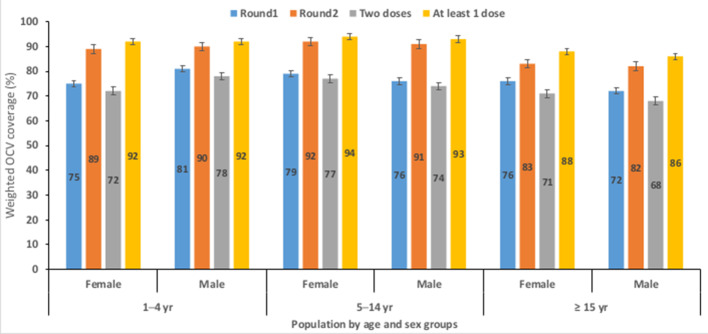
Overall weighted OCV coverage by age and sex groups in the six LGAs combined. Excepting the ‘At least 1 dose’, coverage for the age group 1 to 4 years (yr) old female group was higher than for males of same age group. For children and adults of age 5 to 14 and 15+ years old, coverage was higher among the female than males, respectively. LGAs, Local Government Areas; OCV, oral cholera vaccine.

The card-verified coverage was higher among males 57% (95% CI: 52 to 62) than among females 53% (95% CI: 50 to 59) during second round (online supplementary S3 table A). Among the 1- to 4-year-old children (excepting the ‘At least 1 dose’), the vaccination coverage was lower for females than males while the reverse is true among the 5 to 14 and ≥15-year-old age groups (figure 3, online supplementary S2 figure).

10.1136/bmjgh-2020-002431.supp6 Supplementary data

##### *LGA specific:* Maiduguri, Jere, Konduga, Mafa, Dikwa and Monguno

Overall, complete vaccination ranged from 68% (95% CI: 46 to 86) among those living in Maiduguri to 87% (95% CI: 74 to 95) among those living in Dikwa; the two dose coverage 51% (95% CI: 20 to 82) was lowest among males of working age (≥15 year) group in Maiduguri compared with the rest of the LGAs (online supplementary S3 table B, S3 figure).

10.1136/bmjgh-2020-002431.supp7 Supplementary data

##### Logistic regression of vaccine coverage

In keeping with our secondary objective to compare coverage by sex and age groups, ORs for coverage were computed using two models namely an All LGA and an LGA-specific model (table 2, online supplementary S4 table). Overall, there were no significant differences in crude coverage in any round by sex even after adjusting for age group.

10.1136/bmjgh-2020-002431.supp8 Supplementary data

**Table 2:** Selected LGA ORs of two logistic regression model fits to binary outcome crude coverage

Level	OR (95% CI)		
	Round 1	Round 2	Two doses
**(A) All LGA model**			
Unadjusted*	─	─	─
Adjusted for age†	─	─	─
Modified by age‡	─	─	─
1–4 Female	1.0 (ref)	1.0 (ref)	1.0 (ref)
Male	**1.41 (1.06 to 1.88**)	1.08 (0.73 to 1.59)	**1.39 (1.06 to 1.83**)
≥15 Female	1.0 (ref)	1.0 (ref)	1.0 (ref)
Male	**0.80 (0.64 to 0.98**)	0.95 (0.78 to 1.67)	0.86 (0.70 to 1.06)
**(B) LGA-specific model**			
Maiduguri			
Adjusted for age†			
Female	1.0 (ref)	1.0 (ref)	1.0 (ref)
Male	**0.63 (0.43 to 0.94**)	0.67 (0.45 to 1.00)	0.68 (0.47 to 1.00)
Modified by age‡			
≥15 Female	1.0 (ref)	1.0 (ref)	1.0 (ref)
Male	**0.40 (0.22 to 0.71**)	0.62 (0.38 to 1.01)	**0.50 (0.29 to 0.93**)
Jere			
Modified by age‡			
1–4 Female	1.0 (ref)	1.0 (ref)	1.0 (ref)
Male	**1.95 (1.24 to 3.08**)	1.17 (0.69 to 1.98)	**2.04 (1.34 to 3.12**)
5–14 Female	1.0 (ref)	1.0 (ref)	1.0 (ref)
Male	**0.58 (0.41 to 0.84**)	0.63 (0.34 to 1.17)	**0.58 (0.41 to 0.83**)
Mafa			
Unadjusted*			
Female	1.0 (ref)	1.0 (ref)	1.0 (ref)
Male	**1.31 (1.07 to 1.59**)	1.27 (0.94 to 1.73)	1.23 (1.00 to 1.51)
Adjusted for age†			
Female	1.0 (ref)	1.0 (ref)	1.0 (ref)
Male	**1.24 (1.02 to 1.51**)	1.16 (0.83 to 1.62)	1.18 (0.95 to 1.46)
Modified by age‡			
5–14 Female	1.0 (ref)	1.0 (ref)	1.0 (ref)
Male	1.50 (0.95 to 2.36)	1.64 (0.92 to 2.96)	**1.50 (1.04 to 2.16**)
Konduga			
Unadjusted*			
Female	1.0 (ref)	1.0 (ref)	1.0 (ref)
Male	0.94 (0.74 to 1.21)	**1.43 (1.04 to 1.95**)	0.99 (0.74 to 1.14)

ORs and CIs were estimated using survey-weighted logistic regression. ORs in bold show statistically significant difference. The odds of an outcome is the ratio of the probability the outcome occurs to the probability the outcome does not occur. An OR compares the relative odds of an outcome between two groups. In our case, our outcome is vaccine coverage, so the ORs compare the odds of vaccine coverage between two groups. Specifically, we report ORs of vaccine coverage comparing males to females. We interpret these ORs as follows: OR=1 means no difference in the odds of vaccine coverage between males and females; OR >1 means males have a higher odds of vaccine coverage than females; OR <1 means males have a lower odds of vaccine coverage than females.

*Model was unadjusted for any covariates.

†Model was adjusted for age group.

‡Model included an interaction between age group and sex to allow age to modify the effect of sex on crude coverage.

(A), All LGA model; (B), LGA-specific model; crude coverage, card-verified plus self-reported coverage; LGA, Local Government Area; (ref), reference group.

When we considered allowing the effect of sex to be modified by age, the odds of crude coverage in **first round** were 41% higher for males compared with females in the 1 to 4 years age group, but 20% lower for males compared with females among the 15+ years age group (table 2A, online supplementary S4 table A). Statistically significant sex and age modified differences for coverage were found for the six LGAs during first, second and for complete dose (table 2B, online supplementary S4 table B).

### Adverse events following immunisation

One focus of the survey was to document AEFIs among the population vaccinated in both rounds. Overall, 1.2% reported having fallen sick after the first round of immunisation and 1.1% after the second round; the majority of those who reported the ill heath were from Mafa (3%) (online supplementaryS5 table A). Of the symptoms reported as an AEFI, the most common were fever (50% and 34%), headache (17% and 22%) and diarrhoea (17% and 21%) during first and second rounds, respectively (online supplementary S5 table B). The onset of symptoms ranged from immediately (12% and 17%) to greater than 3 days (10% and 5%) after taking OCV in first and second rounds, respectively (online supplementary S5 table C). The actions taken as remedy to mitigate AEFI symptoms ranged from ‘did nothing’ (30%, second round) to consulting traditional healers (2%, first round) (online supplementary S5 table D).

10.1136/bmjgh-2020-002431.supp9 Supplementary data

### Reasons for vaccination and non-vaccination

As part of this survey, interviewers assessed reasons for vaccination (table 3A) as well as non-vaccination (table 3B) for the first and second rounds of campaign for all LGAs combined and for specific LGAs. About 75% (95% CI: 68 to 81) of the target population reportedly took OCV during both rounds of vaccination primarily to protect themselves from cholera (table 3A); the least common reason for having taken the vaccine was free offer. For those who missed the vaccine, especially during round 2 (all LGAs combined), travelling/working was reportedly the main reason (table 3B); the least common reason for non-vaccination was discouragement from community members. The decision to take or not take the vaccine differed depending on the specific LGA (table 3A and B).

**Table 3:** Factors that guided decision to take or not take the vaccine

Reasons for	Maiduguri	Jere	Konduga	Mafa	Dikwa	Monguno	All LGAs
	(95% CI)	(95% CI)	(95% CI)	(95% CI)	(95% CI)	(95% CI)	(95% CI)
(A) Vaccination/%							
Protect from cholera R1	71 (48 to 88)	74 (56 to 89)	65 (43 to 83)	75 (64 to 83)	63 (39 to 83)	89 (81 to 94)	75 (68 to 81)
Protect from cholera R2	71 (52 to 86)	73 (58 to 85)	66 (45 to 84)	73 (64 to 81)	63 (40 to 82)	86 (76 to 93)	74 (68 to 80)
Convinced by campaigners R1	26 (14 to 39)	29 (20 to 40)	35 (20 to 54)	30 (21 to 41)	52 (34 to 71)	29 (16 to 46)	32 (27 to 38)
Convinced by campaigners R2	31 (15 to 51)	27 (18 to 38)	34 (19 to 50)	32 (22 to 43)	51 (31 to 70)	27 (15 to 43)	31 (26 to 38)
Convinced by CHW R1	17 (6 to 36)	19 (12 to 27)	11 (5 to 21)	15 (8 to 25)	50 (32 to 68)	27 (14 to 43)	22 (18 to 28)
Convinced by CHW R2	14 (5 to 31)	17 (11 to 24)	11 (5 to 21)	15 (8 to 25)	48 (29 to 67)	27 (15 to 43)	22 (17 to 27)
Convinced by local leader R1	15 (5 to 35)	20 (12 to 29)	8 (3 to 15)	3 (2 to 5)	46 (30 to 63)	7 (2 to 16)	16 (12 to 21)
Convinced by local leader R2	15 (4 to 32)	18 (11 to 26)	7 (3 to 13)	4 (3 to 6)	41 (25 to 59)	6 (2 to 13)	15 (11 to 19)
Followed others R1	4 (2 to 9)	11 (6 to 18)	5 (2 to 9)	18 (11 to 27)	39 (24 to 56)	20 (11 to 34)	15 (12 to 20)
Followed others R2	5 (2 to 12	11 (7 to 18)	6 (2 to 11)	19 (12 to 27)	37 (21 to 55)	22 (12 to 34)	16 (12 to 20)
Recommended by doctor R1	11 (4 to 24)	7 (3 to 13)	7 (3 to 11)	1 (0.3 to 3)	0	0	9 (6 to 12)
Recommended by doctor R2	12 (4 to 24)	6 (3 to 12)	7 (4 to 13)	1 (0.4 to 3)	23 (11 to 41)	5 (1 to 14)	8 (6 to 12)
Free offer R1	6 (1 to 18)	3 (1 to 6)	2 (0.4 to 4)	12 (7 to 17)	1 (0.2 to 1.2)	18 (10 to 30)	7 (5 to 11)
Free offer R2	5 (1 to 15)	5 (2 to 10)	1 (0.4 to 4)	13 (8 to 18)	1 (0.2 to 1.2)	19 (11 to 30)	8 (5 to 12)
(B) Non-vaccination/%							
Travel/work R1	13 (2 to 35)	28 (19 to 38)	27 (16 to 40)	40 (20 to 61)	49 (26 to 72)	47 (33 to 63)	32 (25 to 40)
Travel/work R2	18 (4 to 43)	36 (25 to 48)	57 (29 to 82)	28 (14 to 48)	51 (31 to 71)	65 (41 to 85)	39 (29 to 49)
Not aware of campaign R1	70 (37 to 92)	38 (24 to 53)	40 (20 to 63)	37 (15 to 64)	15 (1 to 50)	12 (5 to 22)	36 (24 to 48)
Not aware of campaign R2	55 (21 to 85)	25 (14 to 39)	6 (2 to 15)	39 (18 to 64)	0	5 (1 to 13)	26 (15 to 40)
Not aware of schedule R1	53 (13 to 90)	17 (12 to 24)	20 (7 to 40)	26 (6 to 60)	3 (0.4 to 12)	10 (3 to 23)	22 (11 to 34)
Not aware of schedule R2	41 (7 to 85)	8 (4 to 13)	3 (0.4 to 10)	27 (7 to 57)	7 (1 to 21)	4 (1 to 9)	15 (6 to 31)
Vaccinators did not show up R1	4 (0.7 to 13)	8 (3 to 14)	9 (4 to 17)	7 (3 to 12)	25 (13 to 39)	4 (1 to 7)	7 (5 to 10)
Vaccinators did not show up R2	7 (2 to 19)	15 (8 to 24)	22 (7 to 47)	9 (5 to 15)	24 (7 to 48)	7 (2 to 16)	13 (9 to 18)
Afraid of side effects R1	3 (2 to 5)	1 (0.2 to 5)	2 (0.4 to 4)	1 (0.1 to 2)	0	19 (12 to 29)	6 (3 to 10)
Afraid of side effects R2	2 (0.3 to 5)	3 (0.8 to 8)	2 (0.3 to 5)	1 (0.2 to 4)	0	14 (5 to 27)	4 (2 to 7)
OCV does not help R1	1 (0.1 to 3)	3 (1 to 7)	15 (1 to 50)	9 (4 to 18)	5 (0.4 to 20)	7 (2 to 14)	5 (3 to 9)
OCV does not help R2	3 (2 to 6	3 (1 to 6)	2 (0.2 to 8)	10 (3 to 22)	10 (1 to 35)	5 (1 to 15)	4 (3 to 6)
Time not convenient R1	3 (0.6 to 10)	5 (1 to 13)	7 (0.6 to 25)	3 (1 to 7)	2 (0.1 to 7)	2 (0.5 to 5)	4 (2 to 7)
Time not convenient R2	8 (2 to 22)	12 (7 to 19)	6 (2 to 15)	9 (3 to 19)	6 (0.2 to 31)	2 (0.4 to 4)	8 (6 to 12)
Discouraged by community R1	2 (0.1 to 8)	1 (0.1 to 3)	0.1 (0 to 0.4)	0.4 (0.1 to 2)	0	0	1 (0.1 to 2)
Discouraged by community R2	3 (0.1 to 15)	1 (0.1 to 5)	0	1 (0.1 to 3)	0	0.1 (0 to 0.6)	26 (15 to 39)

(R1) round 1 and (R2) round 2 factors influencing decision varied by round of vaccination and LGA.

CHW, community health worker; OCV, oral cholera vaccine.

### Campaign information sources and vaccinee’s experience with vaccinators

Among the respondents 15 years and over recruited in this survey, information about the OCV campaigns were through a variety of sources. The four most common information sources of the campaign were word of mouth from neighbour, town or village crier, announcements inside IDP camps and relatives (table 4A). An overwhelming majority of the survey respondents felt that vaccinators treated them with respect while just a tiny minority felt otherwise (table 4B). Furthermore, on the question of whether or not the campaign team addressed all respondents’ concerns about the vaccine, majority did not raise or ask any questions while others felt that their concerns were fully (‘Yes’), partially (‘Only partially’) or not (‘No’) addressed (table 4C).

**Table 4:** Campaign information sources and vaccinator behaviour

Measures	Maiduguri	Jere	Konduga	Mafa	Dikwa	Monguno	All LGAs
	(95% CI)	(95% CI)	(95% CI)	(95% CI)	(95% CI)	(95% CI)	(95% CI)
(A) Information sources/%							
Neighbours	58 (36 to 79)	39 (29 to 49)	42 (26 to 61)	43 (28 to 59)	57 (32 to 79)	56 (41 to 70)	48 (42 to 55)
Town/village crier	18 (6 to 38)	37 (27 to 48)	24 (12 to 41)	42 (23 to 63)	57 (39 to 74)	41 (27 to 57	37 (31 to 43)
IDP Camp	19 (7 to 40)	36 (28 to 44)	38 (24 to 53)	19 (11 to 29)	49 (31 to 67)	31 (17 to 48)	32 (27 to 38)
Relative	17 (11 to 26)	21 (15 to 29)	25 (14 to 39)	15 (6 to 27)	33 (20 to 47)	24 (29 to 56)	27 (22 to 32)
Radio	23 (9 to 44)	7 (3 to 13)	5 (2 to 10)	8 (2 to 20)	24 (13 to 39)	2 (0.5 to 6)	10 (7 to 14)
Market	3 (0.8 to 6)	2 (0.4 to 4)	2 (0.4 to 7)	1 (0.4 to 2)	26 (14 to 43)	2 (1 to 5)	5 (3 to 7)
Government official	3 (0.4 to 8)	4 (1 to 9)	5 (0.6 to 18)	0.4 (0.1 to 1)	7 (3 to 15)		3 (2 to 5)
Television	11 (2 to 33)	0.2 (0.1 to 0.6	1 (0.1 to 2)	1 (0.2 to 4)	6 (3 to 12)	0.03 (0.0 to 0.2)	2 (0.8 to 6)
Social media	0.07 (0.1 to 0.2)	0.2 (0.1 to 0.7)	0.2 (0.1 to 0.5)	0	6 (2 to 13)	0	1 (0.3 to 2)
Newspaper	0.3 (0.1 to 0.9)	0.01 (0.0 to 0.1)	0.03 (0.0 to 0.2)	0	4 (2 to 10)	0	1 (0.2 to 1)
(B) Vaccine team respect/%							
Yes	95 (83 to 99)	98 (96 to 99)	98 (96 to 99)	99 (98 to 100)	99 (98 to 100)	92 (87 to 95)	96 (95 to 98)
No	0	0	0	0	0	0	0
(C) Answered all questions/%							
Did not ask questions	38 (18 to 61)	54 (41 to 66)	48 (30 to 67)	54 (43 to 65)	29 (12 to 52)	33 (21 to 47)	43 (36 to 50)
Yes	40 (17 to 66)	31 (22 to 42)	30 (17 to 46)	42 (30 to 54)	63 (43 to 81)	33 (20 to 49)	37 (31 to 44)
Only partially	14 (2 to 39)	9 (4 to 16)	7 (3 to 12)	2 (0.7 to 6)	4 (2 to 8)	23 (14 to 36)	12 (8 to 17)
No	9 (3 to 20)	6 (3 to 10)	15 (7 to 28)	2 (0.8 to 3)	3 (0.7 to 9)	10 (6 to 17)	8 (6 to 10)

Information in the table was not collected with respect to round of campaign.

IDP, Internally Displaced Person; LGAs, Local Government Areas.

## Discussion

This survey documented the response to a cholera outbreak in the context of a humanitarian emergency caused by Boko Haram, found that mass immunisations with OCV within IDP camps and surrounding settlements located in urban and rural areas was highly successful. The civil unrest and the 16 August 2017 cholera outbreak in Muna Garage satisfied one of the three contexts in which the vaccines are requested from the global stockpile.^27^ As such, the reactive mass OCV campaigns in IDP camps and surrounding villages in the six LGAs in Borno were in line with WHO’s policy for emergency use of OCV to halt an active cholera outbreak,^28 29^ and high vaccine coverage was reached.^15 30^ The high coverage highlights the acceptability^31 32^ of OCV in high risk populations in camps and conflict areas in Borno, which is consistent with findings in similar reactive settings.^12 13^ However, high coverage might not necessarily underscore vaccine acceptability. Rather, as the campaigns occurred amidst an ongoing outbreak, the high coverage could be the result of people taking the vaccine because they were frightened by cholera deaths they saw in the community.^10^ This explanation is plausible as we found that 75% (95% CI: 68 to 81) got vaccinated to protect from cholera during first round with a downward trend during second round (table 3A). Still, a full coverage of 73% (95% CI: 68 to 77), though higher compared with other reactive settings,^33–37^ is below the 80% target recommended by WHO to prevent outbreaks;^23^ in addition, this rate could not be confirmed through vaccination cards. This creates room for pockets of populations without OCV protection, thus risking cholera outbreaks. Given the history of vaccine hesitancy in Borno,^38^ we agree with Luquero *et al.*^39^ that qualitative studies are needed to access the behavioural determinants of OCV acceptability in Borno in a preemptive context.

Data of administrative coverage were 105% and 99% during first and second rounds, respectively. As such our survey coverage results does not corroborate the administrative coverages. The 105% administrative coverage during first round suggests that more people were vaccinated than initially targeted. This is very likely because IDPs were continually added to the camp populations as they fled their villages from Boko Haram. Further, the administrative estimates, computed by dividing the number of vaccinations by the number of people eligible for vaccination, could be inaccurate, especially if good records are not kept during vaccination, and if population estimates were wrong. Our survey relied on house-to-house interviews and are less affected by errors in population estimates compared with administrative coverage estimates. With the later, the mean household size of 3.0 persons we found in this study is smaller, as compared with the national average of 4.7 persons obtained from Nigeria Demographic and Health Survey (DHS).^21^ Also the median age of 12 years (IQR=25) we found is far less than the national median of 17.9 years.^40^ We do not understand the reasons for these discrepancies. Perhaps the difficult security context in Borno played a role, as surveyors reported consoling respondents who reported losing household members to Boko Haram.

Importantly, we found that working age (≥15 years) males had lower coverage compared with females of the same age group, and that the difference was statistically significant when we considered effect modification by age group. This finding is consistent with other studies,^30 36–38^ which have found lower coverage in male adults compared with females. In addition, females were more likely to retain their vaccination cards and present them on request during interviews. Still, we found that non-vaccination was predominantly linked with absence during campaigns, which is consistent with the literature.^39 41^ As the campaign used fixed sites and house-to-house strategies, these might have favoured women who tend to spend more time at home compared with men who spend much of their time working outside of the home. Future campaigns should device innovative strategies to target working age men, who are also highly mobile.

During the campaigns, there were no AEFIs reported; however, we identified a low rate of AEFIs, consisting primarily of mild symptoms with symptoms starting quickly after the vaccination or up to 3 days after taking vaccine. The fact that only mild symptoms were reported speaks to the safety of OCV as has been documented elsewhere.^37 42^ Some respondents did nothing against the symptoms, and others self-medicated at home or pharmacy. Although OCV was found to be safe, the programmes should have plans in place to manage these should they occur.

As OCV was used in Borno for the first time, it was important that the campaign address respondents concerns. We laud the efforts of the campaign team as large majority of respondents felt that the team treated them with respect. It is not surprising that campaign awareness was mostly through neighbours and village criers, and rarely through mass media, as very few people in the camps and surrounding villages had access to electricity. This underscores the importance of bottom up approach in social mobilisation efforts in resource poor settings.

This study has important limitations. The post-coverage survey planned to visit 30 clusters in each LGA, but we missed one cluster in Maiduguri. Of the 5400 household planned to be visited, contacts were made to 5275 yielding a contact rate of 97.63%. In view of the insecurity posed by Boko Haram armed insurgency in Borno at the time of this survey,^5 6^ this contact rate is highly encouraging and reflects the resolve of dedicated data collectors who defied all odds for the success of the survey. Due to the difficult security context we were unable to involve patients/public in the study, involvement of which would have enriched the quality of our findings. As vaccination cards were not issued during first round, we were not able to confirm vaccination status for that round. Still, despite card issuance during second round, we were unable to confirm vaccination status for 12.98% of respondents due to missing cards. Although the manufacturer recommends 14 days apart between the first and second round with Shanchol,^43^ the second round was administered >80 days from the first. As a result, we cannot rule out recall bias in our coverage estimates. Despite these limitations, this survey used rigorous methodology in study design, sampling that ensured representative samples in all LGAs, adequate training of surveyors that ensured data quality using CAPI during data collection as well as weighted analysis that accounted for missing data and the complex survey design. To this end, we think that the results of this coverage survey can be generalised to the target population in the six LGAs surveyed.

In sum, the OCV campaign in Borno was successfully implemented in 5 days, which was able to provide vaccine to about one million people using house-to-house and fixed site strategies in the face of Boko Haram armed insurgency. The coverage rate was high, although working age men had lower coverage than women. Low coverage in working age men coupled with the fact that absence during campaign was the main reason for non-vaccination underscore the need to explore alternative strategies to administer the vaccine in mobile subpopulations. Conducting pre-campaign qualitative research to understand behavioural determinants of vaccine acceptability and how to reach mobile subpopulations should be the starting point for exploring the alternative vaccine distribution strategies. However, as OCV is not a long-term remedy to cholera and only bridges the gap between emergency response and long-term cholera control, additional actions will be needed to make investments in water, sanitation and hygiene infrastructure.
