# Supplementary material for: The reactive vaccination campaign against cholera emergency in camps for internally displaced persons, Borno, Nigeria, 2017: a two-stage cluster survey
Source: BMJ Glob Health. 2020 Jun 29;5(6):e002431. doi: 10.1136/bmjgh-2020-002431 (PMC7326259; doi:10.1136/bmjgh-2020-002431)
Supplement: Supplementary data [file bmjgh-2020-002431supp001.pdf]

S1 Table. Form ward: Sample cluster selection using probability proportional to population size

		Jere LGA*				
**Ward**	**Camp**	**Target Pop**	**Cum Pop**	**Cluster tally**	**Clusters selected**	**Cluster ID**
Old Maiduguri	Ajari old Maiduguri	3744	3744	1,	1	1
Dala	Bulamari	317	4061			
Dusuman	Custom House1	5630	9691	1,	1	2
Dusuman	Custom House 2	3429	13120	1,	1	3
Dala	Dala Foi	15797	28917	1,1,1,1,	4	4,5,6,7
Mairi	Dalori Camp	28942	57859	1,1,1,1,1,1,	6	8,9,10,11,12,13
Old Maiduguri	Faria Buzu Camp	243	58102			
Old Maiduguri	Faria Gidan Block	8729	66831	1,1,	2	14,15
Dusuman	Farm Center	13384	80215	1,1,1,	3	16,17,18
Dala	Fatia Kurti	212	80427			
Old Maiduguri	Hursu Bashir	2983	83410	1,	1	19
Dala	Kurari	446	83856			
Old Maiduguri	Lambu	1990	85846	1,	1	20
Old Maiduguri	Madinatu	1195	87041			
Old Maiduguri	Miskin	1897	88938			
Dusuman	Muna Da'ali 2	229	89167			
Dusuman	Muna Da'alti 1A	562	89729			
Dusuman	Muna Da'alti 1B	141	89870	1,	1	21
Dusuman	Muna Da'alti 2A	6311	96181	1,	1	22
Dusuman	Muna Da'alti 2B	390	96571			
Dusuman	Muna Da'alti 3	141	96712			
Dusuman	Muna Ethiopia gulumba	493	97205			
Dusuman	Muna garage elbarnawi	19975	117180	1,1,1,1,1	5	23,24,25,26,27
Dusuman	Muna Kori bula Yalaye	462	117642			
Dusuman	Muna Moforo	2357	119999			
Dusuman	Muna Primary	442	120441	1,	1	28
Dusuman	Musari Camp	1078	121519			
Mairi	Uniguess	3891	125410	1,	1	29
Mairi	Vetenary village	5854	131264	1,	1	30

***OCV sample cluster selection for Jere LGA; the same process was done to select 30 clusters for the rest of the five LGAs**

Ngwa MC, *et al. BMJ Global Health* 2020; 5:e002431. doi: 10.1136/bmjgh-2020-002431
