# Supplementary material for: The reactive vaccination campaign against cholera emergency in camps for internally displaced persons, Borno, Nigeria, 2017: a two-stage cluster survey
Source: BMJ Glob Health. 2020 Jun 29;5(6):e002431. doi: 10.1136/bmjgh-2020-002431 (PMC7326259; doi:10.1136/bmjgh-2020-002431)
Supplement: Supplementary data [file bmjgh-2020-002431supp002.pdf]

S2 Table: Survey Questionnaire

Oral Cholera Vaccine, Borno State, Nigeria Coverage Survey Questionnaire, February 2018

Question and Options
English
001. Village/cluster name
text
002. Interviewer Number
integer
002a. Interviewer Name
text
002b. Supervisor Number
integer
002c. Supervisor Name
text
003. Date of interview
date
003a. Time of interview
time
004. Household ID Number
integer
005. Informed consent given?
No
Yes
006. If No and family not at home
Information from neighbor
Information from local leader
007. Number of members living in the household
integer
007a. How many household members available now?
integer
008. Number of members living in the household by age strata
1-4 years
5-14 years
15 years and above
008a. How many household members available now by age strata?
1-4 years
5-14 years
15 years and above
009. Household member ID
integer
101. Age of respondent
integer
102. Sex of respondent
Male
Female

Ngwa MC, *et al. BMJ Global Health* 2020; 5:e002431. doi: 10.1136/bmjgh-2020-002431

103. Who responded to the interview?
Self
Parent/Caretaker

FIRST ROUND

104. Received Dose 1?
Yes, Card seen
Yes, Card not seen
Dose not received

104a. Date of Dose 1 as reported on vaccination card (dd/mm/yyyy)
date

104a1. Take a picture of the vaccination card
image

104b. Where did you take your 1st dose of vaccine
Home
Health facility
Outreach site
Camp
Market
School
Other

104b1.Other places where dose 1 was taken
text

104c. Reason for taking Dose 1
To protect from cholera (self-motivated)
Motivated by campaigners that vaccine is safe
Suggested by doctor that vaccine is safe
Suggested by local leader that vaccine is safe
The Community Health Worker assured that the vaccine is safe
Followed others in the community that said vaccine is safe
Free offer
Other

104c1. Other Reason for taking Dose 1
text

104d. Reason for not taking Dose 1
Afraid of side effects
Heard that the taste is bad
Time was not convenient
Not aware of the vaccination campaign
Not aware of vaccination schedule
Vaccination center is far away from home
Discouraged by others in the community
Bad experience with vaccinators
Out of town/travelling/Working
It does not help/Not interested in vaccine
Other

104d1. Other Reason for not taking Dose 1
text

104e.Did you get sick after receiving the 1st dose of the OCV?

Yes
No (skip to 105)
Don't know (skip to 105)

104e1.If yes, did you have the symptoms before you took the OCV?

Yes (skip to 105)
No
Don't know (skip to 105)

104e2. Which main symptom did you have?

Nausea
Diarrhea
Fever
Vomiting
Abdominal pain
Headache
Rash
Other

104e3. Specify other symptom

text

104e4. How long after receiving the OCV did you start experiencing the symptom?

Immediately
Less than 30 mins
After 1 hour
The same day (after 12 hours)
Between 1 and 2 days (Between 24 and 48 hours)
More than 3 days (More than 48 hours)
Don't know

104e5. What did you do after experiencing the above symptom?

Nothing
Went to health facility
Saw a doctor
Community Health Workers
Traditional healer
Self-medication at home
Self-medication at pharmacy
Don't know
Other

104e6. If other, please specify

text

SECOND ROUND

105. Received Dose 2?

Yes, Card seen
Yes, Card not seen
Dose not received

105a. Date of Dose 2 as reported on vaccination card (dd/mm/yyyy)

date

105b. Where did you take your 2nd Dose of vaccine

Ngwa MC, *et al. BMJ Global Health* 2020; 5:e002431. doi: 10.1136/bmjgh-2020-002431

Home
Health facility
Outreach site
Camp
Market
School
Other

105b1.Other places where dose 2 was taken
text

105c. Reason for taking Dose 2
To protect from cholera (self-motivated)
Motivated by campaigners that vaccine is safe
Suggested by doctor that vaccine is safe
Suggested by local leader that vaccine is safe
The Community Health Worker assured that the vaccine is safe
Followed others in the community that said vaccine is safe
Free offer
Other

105c1. Other Reason for taking Dose 2
text

105d. Reason for not taking Dose 2
Afraid of side effects
Heard that the taste is bad
Time was not convenient
Not aware of the vaccination campaign
Not aware of vaccination schedule
Vaccination center is far away from home
Discouraged by others in the community
Bad experience with vaccinators
Out of town/travelling/Working
It does not help/Not interested in vaccine
Other

105d1. Other Reason for not taking Dose 2
text

105d. Where did you take your 2nd dose of vaccine
Home
Health facility
Outreach site
Camp
Market
School
Other

105d1.Other places where dose 2 was taken
text

105e.Did you get sick after receiving the 2nd dose of the OCV?
Yes
No

Don't know

105e1.If yes, did you have the symptoms before you took the OCV?

Yes

No

Don't know

105e2. Which main symptom did you have?

Nausea

Diarrhea

Fever

Vomiting

Abdominal pain

Headache

Rash

Other

105e3. Specify other symptom

text

105e4. How long after receiving the OCV did you start experiencing the symptom?

Immediately

Less than 30 mins

After 1 hour

The same day (after 12 hours)

Between 1 and 2 days (Between 24 and 48 hours)

More than 3 days (More than 48 hours)

Don't know

105e5. What did you do after experiencing the above symptom?

Nothing

Went to health facility

Saw a doctor in community

Saw community Health Workers

Went to traditional healer

Self-medication at home

Self-medication at pharmacy

Don't know

Other

105e6.If other, please specify

text

Campaign information sources and Experience

202. Source of information about the vaccine campaign

Neighbor

Relative / Friend

Village/Town crier

Market

Camp

Radio

TV

Newspaper

Ngwa MC, *et al. BMJ Global Health* 2020; 5:e002431. doi: 10.1136/bmjgh-2020-002431

Social Media
Govt.official
Pharmacy
Other

202a. If other source, please specify
text

202b. Did the vaccination team treat you with respect?
Yes
No

202b1. Did the vaccination team answer all your questions?
Yes
No
Not asked any questions
Don't know

202b2. Did the vaccination team inform you about the second dose?
Yes
No
Already took Dose 2
Don't know

202b3. Were you informed about when to come back for second dose?
Yes
No
Already took Dose 2
Don't know

202b4. Were you informed about where to go for the second dose?
Yes
No
Already took Dose 2
Don't know

GPS Location, standing in front of the main entrance door
Integer

Interviewer's comments
text

Supervisor's comments
text
