# Supplementary material for: The reactive vaccination campaign against cholera emergency in camps for internally displaced persons, Borno, Nigeria, 2017: a two-stage cluster survey
Source: BMJ Glob Health. 2020 Jun 29;5(6):e002431. doi: 10.1136/bmjgh-2020-002431 (PMC7326259; doi:10.1136/bmjgh-2020-002431)
Supplement: Supplementary data [file bmjgh-2020-002431supp003.pdf]

Supporting information

Figure legends

S1 figure: Household survey composition. Insert (A, n= 5,272) describes visited households into 'Family not at home' (orange color); 'No'—respondents who were at home but refused consent (green color); and 'Yes— respondents who consented to the interviews (blue color). (B) Shows the distribution of household sizes of 'Family not at home' (orange color) and consented households (blue color).

S2 figure. Overall crude (A-B) and card-verified (C-D) coverage distribution by age and sex in all six LGAs combined in rounds 1 and 2. (A) Crude coverage in round 2, (B) crude coverage in round 1, (C) card-verified coverage in round 2, (D) card-verified coverage in round 1. Note that (D) vaccination cards were not issued during round 1, and effort to document round 1 vaccination during round 2 was later dropped. Shaded areas show 95% confidence intervals for each gender around the smooth lines. Smoothing method used the 'GAM' model. Dashed lines refer to round 2 vaccination activities while continuous lines represent round 1 immunization activities.

S3 figure. Crude coverage estimate distribution by gender and age in each LGA. Fig shows the contribution of each LGA (Maiduguri, Jere, Konduga, Mafa, Dikwa and Monguno) to the combined coverage. Solid lines indicate crude estimates for round 1 and dashed lines show crude estimates for round 2, respectively. Card-verified coverage is not shown. Shaded areas show 95% confidence intervals for each gender around the smooth lines. Smoothing method used the 'GAM' model.
