# Supplementary figures and images for: The reactive vaccination campaign against cholera emergency in camps for internally displaced persons, Borno, Nigeria, 2017: a two-stage cluster survey
Source: BMJ Glob Health. 2020 Jun 29;5(6):e002431. doi: 10.1136/bmjgh-2020-002431 (PMC7326259; doi:10.1136/bmjgh-2020-002431)

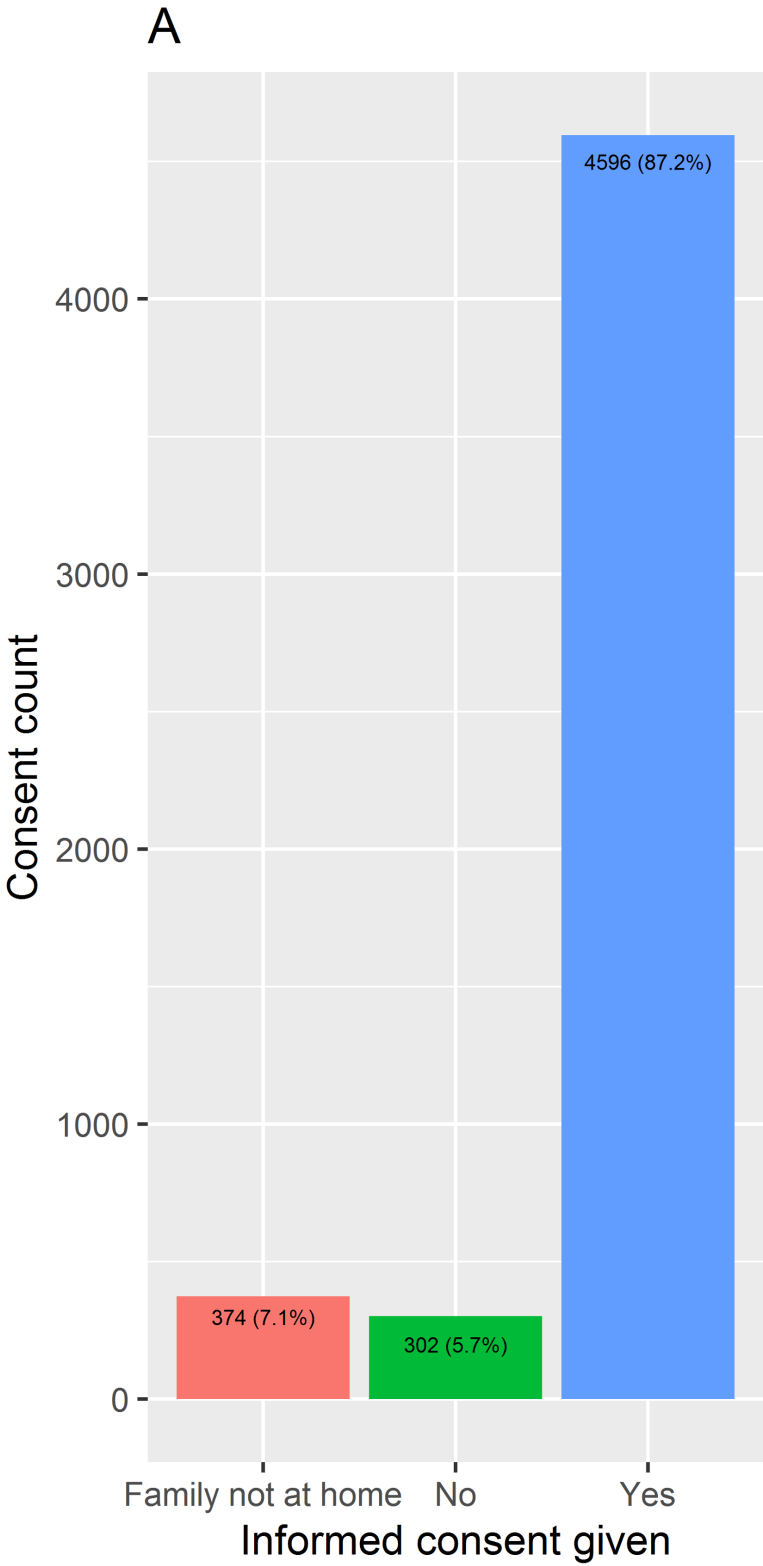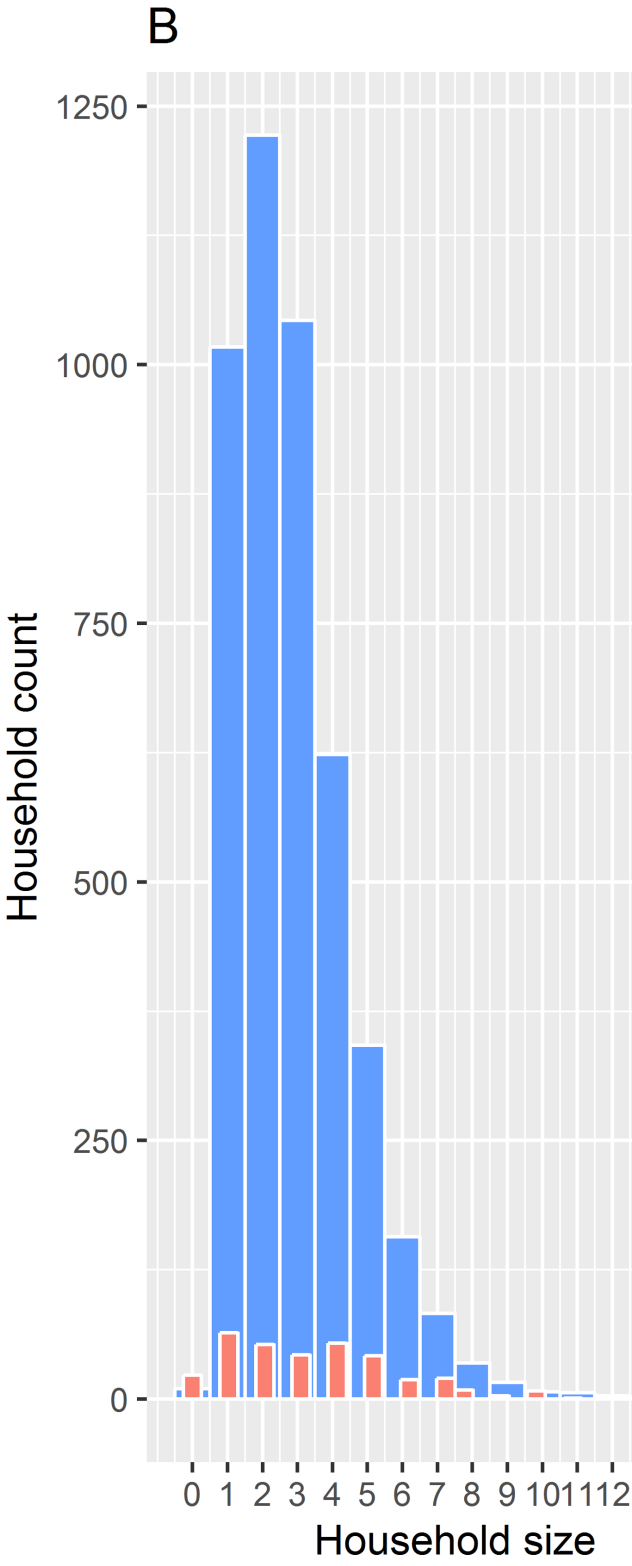

Supplement: Supplementary data [file bmjgh-2020-002431supp004.pdf]

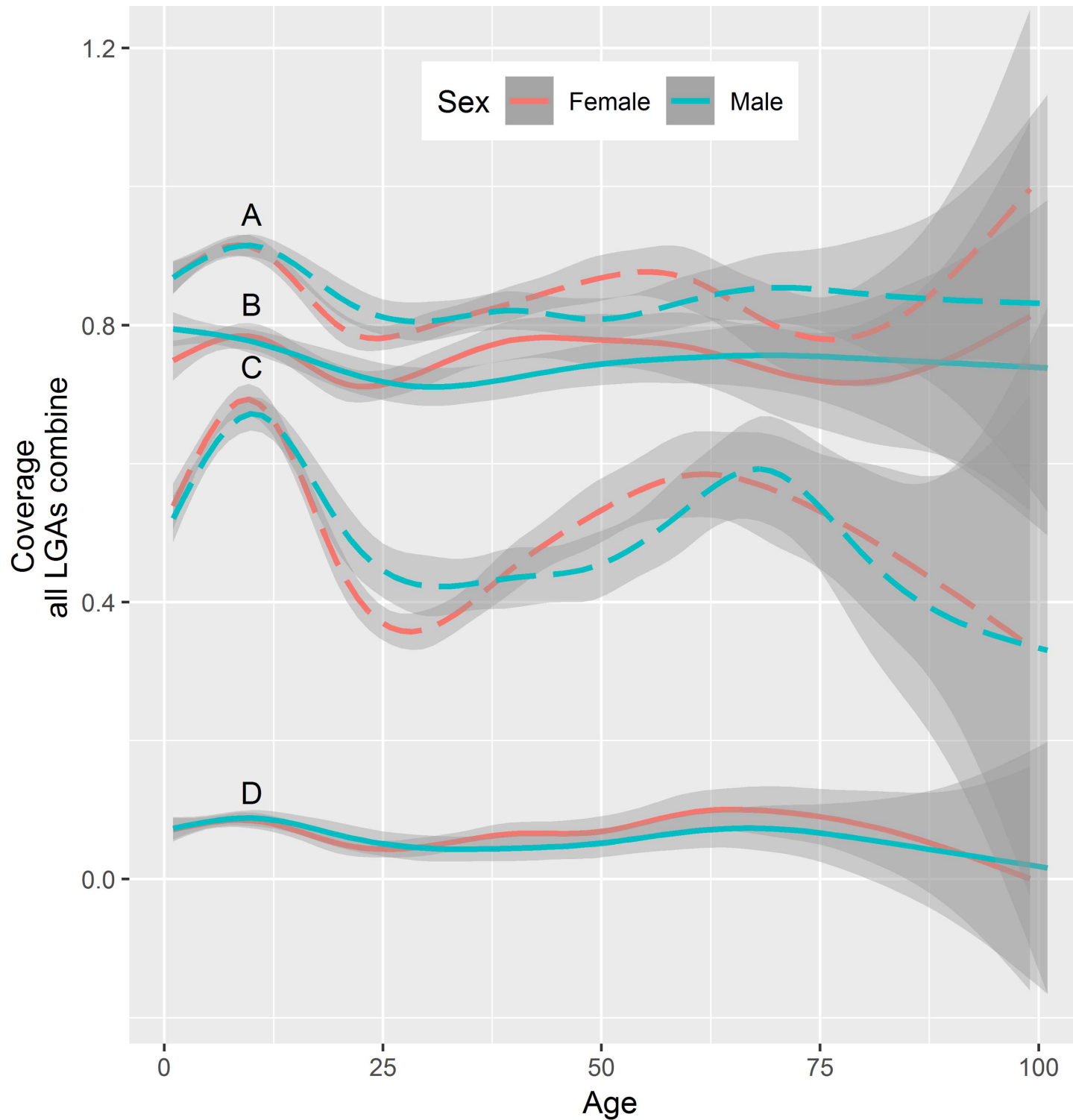

Supplement: Supplementary data [file bmjgh-2020-002431supp006.pdf]

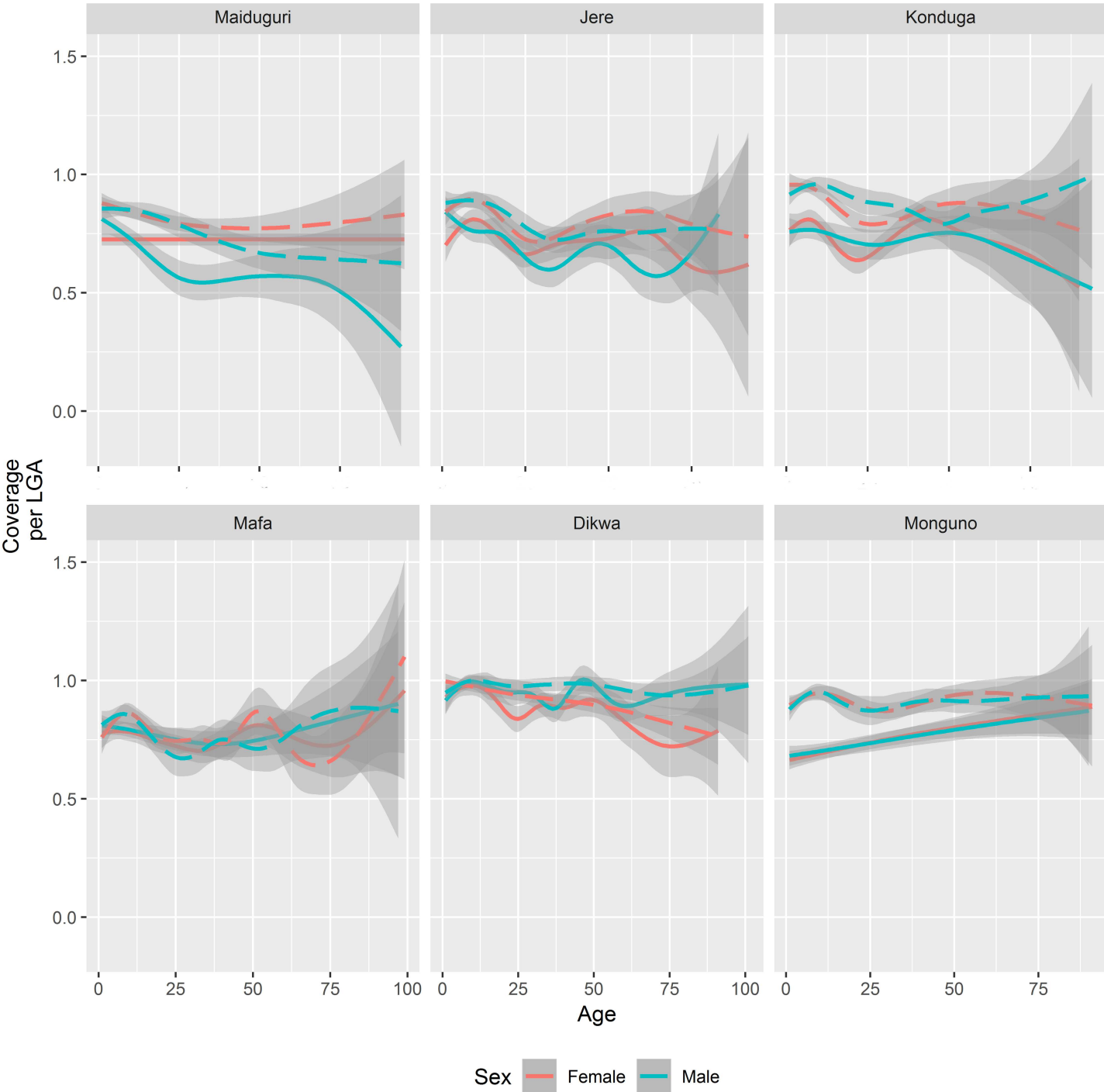

Supplement: Supplementary data [file bmjgh-2020-002431supp007.pdf]
