# Supplementary material for: The reactive vaccination campaign against cholera emergency in camps for internally displaced persons, Borno, Nigeria, 2017: a two-stage cluster survey
Source: BMJ Glob Health. 2020 Jun 29;5(6):e002431. doi: 10.1136/bmjgh-2020-002431 (PMC7326259; doi:10.1136/bmjgh-2020-002431)
Supplement: Supplementary data [file bmjgh-2020-002431supp005.pdf]

S3 Table: Weighted card-verified and crude coverage estimates.

		Weighted coverage estimates					
		Card-verified % [95%CI]		Crude coverage% [95%CI]			
Location		Round 1*	Round 2	Round 1	Round 2	Rounds 1&2	At least 1 dose
**A.** All	LGAs combined						
	Overall	-	55 [50-59]	76 [71-80]	87 [84-89]	73 [68-77]	90 [88-92]
	Sex						
	Female	-	53 [48-58]	77 [72-81]	87 [84-89]	73 [68-77]	90 [88-92]
	Male	-	57 [52-62]	76 [70-80]	87 [84-90]	73 [67-78]	90 [87-93]
	Age group (yr)						
	Female 1-4	-	58 [51-65]	75 [68-81]	89 [85-92]	72 [65-79]	92 [88-95]
	5-14	-	69 [64-74]	79 [73-83]	92 [90-94]	77 [71-82]	94 [92-96]
	≥15	-	43 [39-48]	76 [71-80]	83 [79-86]	71 [66-76]	88 [85-90]
	Male 1-4	-	61 [54-67]	81 [76-86]	90 [86-93]	78 [73-83]	92 [88-95]
	5-14	-	65 [59-71]	76 [71-82]	91 [87-94]	74 [68-79]	93 [90-96]
	≥15	-	49 [43-55]	72 [65-78]	82 [78-86]	68 [61-74]	86 [81-90]
**B.** LGA	specific						
Maiduguri							
	Overall	-	42 [32-52]	72 [49-89]	82 [71-91]	68 [46-86]	87 [75-94]
	Sex						
	Female	-	40 [33-49]	75 [56-89]	84 [75-91]	70 [51-85]	89 [81-94]
	Male	-	43 [29-59]	68 [40-89]	81 [65-91]	65 [38-86]	84 [67-95]
	Age group (yr)						
	Female 1-4	-	38 [26-50]	75 [59-88]	88 [80-94]	72 [55-86]	91 [83-96]
	5-14	-	62 [45-77]	76 [54-92]	91 [82-96]	74 [53-90]	93[85-97]
	≥15	-	29 [21-37]	75 [55-89]	79 [67-88]	68 [48-84]	86 [75-93]
	Male 1-4	-	44 [26-63]	78 [54-93]	85 [66-96]	74 [51-90]	89 [68-98]
	5-14	-	55 [41-69]	75 [50-92]	88 [78-94]	71 [47-89]	92 [83-97]
	≥15	-	31 [15-50]	54 [22-84]	70 [47-87]	51 [20-82]	73 [48-90]
Jere							
	Overall	-	48 [40-56]	73 [64-81]	84 [78-88]	69 [59-77]	88 [83-92]
	Sex						
	Female	-	48 [38-58]	74 [64-82]	84 [78-88]	69 [58-78]	89 [84-93]
	Male	-	48 [41-56]	72 [62-80]	83 [77-89]	68 [59-77]	87 [80-92]
	Age group (yr)						
	Female 1-4	-	47 [38-57]	73 [57-86]	87 [79-93]	69 [53-83]	90 [83-95]
	5-14	-	64 [53-75]	81 [71-89]	91 [86-95]	79 [69-87]	94 [88-97]
	≥15	-	39 [28-50]	70 [59-80]	78 [70-85]	63 [51-74]	85 [78-90]
	Male 1-4	-	56 [44-67]	84 [75-91]	88 [80-94]	82 [73-89]	90 [83-96]
	5-14	-	52 [42-62]	71 [60-81]	87 [78-93]	68 [56-79]	90 [82-95]
	≥15	-	39 [31-48]	64 [52-74]	76 [68-83]	58 [48-69]	82 [73-89]
Konduga							
	Overall	-	51 [42-60]	80 [72-86]	88 [82-93]	77 [69-83]	91 [84-96]
	Sex						
	Female	-	50 [41-60]	80 [71-87]	87 [78-93]	77 [69-84]	90 [80-96]
	Male	-	52 [40-63]	79 [71-86]	90 [86-94]	76 [68-83]	93 [88-96]
	Age group (yr)						
	Female 1-4	-	66 [50-79]	83 [73-90]	92 [85-97]	79 [68-87]	96 [89-99]
	5-14	-	64 [52-75]	83 [74-91]	92 [84-97]	81 [72-88]	94 [86-98]
	≥15	-	40 [32-49]	78 [68-86]	83 [73-90]	74 [64-83]	86 [75-94]
	Male 1-4	-	61 [49-74]	83 [73-91]	95 [89-98]	82 [71-90]	96 [90-99]
	5-14	-	67 [52-80]	84 [75-91]	96 [90-98]	82 [74-89]	97 [92-99]
	≥15	-	36 [22-53]	73 [62-83]	84 [77-89]	69 [57-80]	88 [81-93]
Mafa							
	Overall	-	45 [39-51]	81 [71-89]	86 [76-93]	77 [66-86]	90 [81-95]
	Sex						
	Female	-	43 [36-50]	79 [69-87]	85 [74-92]	76 [64-85]	88 [79-95]
	Male	-	46 [39-53]	83 [74-91]	87 [77-94]	79 [68-88]	92 [84-97]
	Age group (yr)						
	Female 1-4	-	56 [41-70]	83 [73-90]	86 [72-94]	78 [65-88]	90 [80-97]
	5-14	-	58 [47-69]	79 [70-87]	88 [79-94]	76 [66-84]	92 [84-97]
	≥15	-	29 [23-37]	78 [66-88]	82 [69-91]	75 [62-85]	86 [74-94]
	Male 1-4	-	43 [33-53]	86 [72-95]	88 [78-95]	81 [65-92]	94 [87-97]
	5-14	-	62 [52-71]	85 [76-92]	92 [83-97]	82 [72-90]	95 [87-99]
	≥15	-	29 [20-39]	79 [67-89]	81 [65-91]	74 [59-86]	86 [73-94]
Dikwa							

Ngwa MC, *et al. BMJ Global Health* 2020; 5:e002431. doi: 10.1136/bmjgh-2020-002431

	Overall	-	78 [65-87]	87 [75-95]	94 [88-97]	87 [74-95]	94 [88-97]
	Sex						
	Female	-	72 [57-83]	84 [70-93]	91 [84-96]	84 [69-93]	92 [85-96]
	Male	-	86 [74-93]	91 [79-98]	97 [93-99]	91 [79-98]	97 [93-99]
	Age group (yr)						
	Female 1-4	-	94 [76-99]	89 [65-99]	94 [76-100]	89 [65-99]	94 [76-100]
	5-14	-	92 [79-98]	92 [76-99]	99 [97-100]	92 [76-99]	99 [97-100]
	≥15	-	59 [44-73]	80 [68-89]	87 [80-93]	79 [66-89]	88 [82-93]
	Male 1-4	-	86 [70-95]	92 [76-99]	96 [81-100]	91 [74-98]	97 [85-100]
	5-14	-	95 [89-98]	95 [74-100]	100 [100-100]	95 [74-100]	100 [100-100]
	≥15	-	80 [62-92]	89 [79-96]	95 [90-98]	89 [79-96]	95 [90-98]
Monguno							
	Overall	-	69 [58-79]	75 [64-83]	91 [85-95]	72 [61-82]	94 [88-97]
	Sex						
	Female	-	66 [54-77]	75 [63-84]	91 [84-95]	72 [60-83]	94 [87-98]
	Male	-	74 [63-83]	74 [64-82]	91 [85-95]	71 [61-81]	94 [88-97]
	Age group (yr)						
	Female 1-4	-	69 [49-84]	69 [51-83]	90 [77-97]	67 [49-82]	91 [79-98]
	5-14	-	81 [68-90]	69 [54-82]	94 [87-98]	69 [53-82]	95 [88-98]
	≥15	-	58 [47-68]	80 [71-87]	90 [83-95]	76 [65-85]	94 [88-98]
	Male 1-4	-	79 [66-89]	71 [54-85]	90 [81-96]	68 [50-82]	93 [85-98]
	5-14	-	81 [67-91]	71 [59-81]	94 [85-98]	70 [58-81]	94 [85-99]
	≥15	-	65 [53-77]	78 [69-86]	89 [81-95]	74 [63-84]	93 [89-96]

**Vaccination cards were not issued during round 1. (A) Overall weighted coverage estimates in all six LGAs combined. (B) Contribution of each LGA to the combined weighted coverage estimates. Crude coverage = card-verified plus self-reported coverage; CI = Confidence Interval; yr = years. Rounds 1&2, complete vaccination (two doses of vaccine during the two rounds of vaccination). At least 1 dose, for those who received one dose of OCV either in round 1 or round 2.*
