# Supplementary material for: The reactive vaccination campaign against cholera emergency in camps for internally displaced persons, Borno, Nigeria, 2017: a two-stage cluster survey
Source: BMJ Glob Health. 2020 Jun 29;5(6):e002431. doi: 10.1136/bmjgh-2020-002431 (PMC7326259; doi:10.1136/bmjgh-2020-002431)
Supplement: Supplementary data [file bmjgh-2020-002431supp008.pdf]

S4 Table: Odds Ratios (ORs) estimates from two logistic regression model fits to binary outcome variable crude coverage

**Level**			OR (95% CI)	
		Round 1	Round 2	Rounds 1&2
All LGAs		***A. All LGA model***		
	Unadjusted[1]			
	Female	1.0 (ref)	1.0 (ref)	1.0 (ref)
	Male	0.94 (0.82-1.09)	1.04 (0.88-1.24)	0.98 (0.86-1.13)
	Adjusted for age[2]			
	Female	1.0 (ref)	1.0 (ref)	1.0 (ref)
	Male	0.92 (0.78-1.07)	0.95 (0.80-1.13)	0.94 (0.82-1.09)
	Modified by age[3]			
	1-4 Female	1.0 (ref)	1.0 (ref)	1.0 (ref)
	Male	**1.41 (1.06-1.88)**	1.08 (0.73-1.59)	**1.39 (1.06-1.83)**
	5-14 Female	1.0 (ref)	1.0 (ref)	1.0 (ref)
	Male	0.88 (0.71-1.08)	0.84 (0.61-1.17)	0.86 (0.70-1.05)
	≥15 Female	1.0 (ref)	1.0 (ref)	1.0 (ref)
	Male	**0.80 (0.64-0.98)**	0.95 (0.78-1.67)	0.86 (0.70-1.06)
		B. LGA specific models		
1.Maiduguri				
	Unadjusted[1]			
	Female	1.0 (ref)	1.0 (ref)	1.0 (ref)
	Male	0.70 (0.49-1.00)	0.80 (0.55-1.17)	0.76 (0.55-1.07)
	Adjusted for age[2]			
	Female	1.0 (ref)	1.0 (ref)	1.0 (ref)
	Male	**0.63 (0.43-0.94)**	0.67(0.45-1.00)	0.68 (0.47-1.00)
	Modified by age[3]			
	1-4 Female	1.0 (ref)	1.0 (ref)	1.0 (ref)
	Male	1.13 (0.58-2.18)	0.77 (0.29-2.04)	1.08 (0.60-1.94)
	5-14 Female	1.0 (ref)	1.0 (ref)	1.0 (ref)
	Male	0.94 (0.70-1.26)	0.73 (0.54-1.00)	0.85 (0.68-1.07)
	≥15 Female	1.0 (ref)	1.0 (ref)	1.0 (ref)
	Male	**0.40 (0.22-0.71)**	0.62 (0.38-1.01)	**0.50 (0.29-0.93)**
2.Jere				
	Unadjusted[1]			
	Female	1.0 (ref)	1.0 (ref)	1.0 (ref)
	Male	0.90 (0.70-1.17)	0.97 (0.73-1.28)	0.97 (0.74-1.26)
	Adjusted for age[2]			
	Female	1.0 (ref)	1.0 (ref)	1.0 (ref)
	Male	0.84 (0.64-1.09)	0.87 (0.38-1.01)	0.89 (0.67-1.18)
	Modified by age[3]			
	1-4 Female	1.0 (ref)	1.0 (ref)	1.0 (ref)
	Male	**1.95 (1.24-3.08)**	1.17 (0.69-1.98)	**2.04 (1.34-3.12)**
	5-14 Female	1.0 (ref)	1.0 (ref)	1.0 (ref)
	Male	**0.58 (0.41-0.84)**	0.63 (0.34-1.17)	**0.58 (0.41-0.83)**
	≥15 Female	1.0 (ref)	1.0 (ref)	1.0 (ref)
	Male	0.74 (0.49-1.14)	0.82 (0.52-1.31)	0.82 (0.52-1.31)
3.Konduga				
	Unadjusted[1]			
	Female	1.0 (ref)	1.0 (ref)	1.0 (ref)
	Male	0.94 (0.74-1.21)	**1.43 (1.04-1.95)**	0.99 (0.81-1.20)

	Adjusted for age[2]			
	Female	1.0 (ref)	1.0 (ref)	1.0 (ref)
	Male	0.88 (0.69-1.12)	1.24 (0.98-1.58)	0.92 (0.74-1.14)
	Modified by age[3]			
	1-4 Female	1.0 (ref)	1.0 (ref)	1.0 (ref)
	Male	1.03 (0.53-2.02)	1.57 (0.44-5.63)	1.23 (0.59-2.55)
	5-14 Female	1.0 (ref)	1.0 (ref)	1.0 (ref)
	Male	1.04 (0.63-1.73)	1.88 (0.61-5.78)	1.09 (0.72-1.66)
	≥15 Female	1.0 (ref)	1.0 (ref)	1.0 (ref)
	Male	0.78 (0.55-1.11)	1.08 (0.78-1.50)	0.78 (0.55-1.10)
4.Mafa				
	Unadjusted[1]			
	Female	1.0 (ref)	1.0 (ref)	1.0 (ref)
	Male	**1.31 (1.07-1.59)**	1.27 (0.94-1.73)	1.23 (1.00-1.51)
	Adjusted for age[2]			
	Female	1.0 (ref)	1.0 (ref)	1.0 (ref)
	Male	**1.24 (1.02-1.51)**	1.16 (0.83-1.62)	1.18 (0.95-1.46)
	Modified by age[3]			
	1-4 Female	1.0 (ref)	1.0 (ref)	1.0 (ref)
	Male	1.28 (0.71-2.31)	1.20 (0.66-2.18)	1.15 (0.66-1.99)
	5-14 Female	1.0 (ref)	1.0 (ref)	1.0 (ref)
	Male	1.50 (0.95-2.36)	1.64 (0.92-2.96)	**1.50 (1.04-2.16)**
	≥15 Female	1.0 (ref)	1.0 (ref)	1.0 (ref)
	Male	1.05 (0.72-1.53)	0.94 (0.54-1.63)	0.98 (0.64-1.50)
5.Dikwa				
	Unadjusted[1]			
	Female	1.0 (ref)	1.0 (ref)	1.0 (ref)
	Male	1.99 (0.92-4.29)	**2.92 (1.83-4.67)**	2.03 (0.97-4.26)
	Adjusted for age[2]			
	Female	1.0 (ref)	1.0 (ref)	1.0 (ref)
	Male	1.86 (0.89-3.40)	**2.63 (1.66-4.17)**	1.90 (0.93-3.88)
	Modified by age[3]			
	1-4 Female	1.0 (ref)	1.0 (ref)	1.0 (ref)
	Male	1.40 (0.25-7.77)	1.36 (0.13-14.17)	1.26 (0.22-7.00)
	5-14 Female	1.0 (ref)	1.0 (ref)	1.0 (ref)
	Male	1.50 (0.29-7.87)	—	1.50 (0.29-7.87)
	≥15 Female	1.0 (ref)	1.0 (ref)	1.0 (ref)
	Male	1.05 (0.72-1.53)	**2.95 (1.81-4.81)**	**2.19 (1.45-3.31)**
6.Monguno				
	Unadjusted[1]			
	Female	1.0 (ref)	1.0 (ref)	1.0 (ref)
	Male	0.95 (0.73-1.25)	1.00 (0.70-1.41)	0.96 (0.75-1.22)
	Adjusted for age[2]			
	Female	1.0 (ref)	1.0 (ref)	1.0 (ref)
	Male	1.01 (0.76-1.33)	0.96 (0.67-1.37)	0.99 (0.78-1.27)
	Modified by age[3]			
	1-4 Female	1.0 (ref)	1.0 (ref)	1.0 (ref)
	Male	1.11 (0.65-1.88)	1.08 (0.43-2.72)	1.03 (0.63-1.68)
	5-14 Female	1.0 (ref)	1.0 (ref)	1.0 (ref)
	Male	1.08 (0.69-1.69)	0.91 (0.55-1.51)	1.07 (0.69-1.66)
	≥15 Female	1.0 (ref)	1.0 (ref)	1.0 (ref)

Male	0.90 (0.61-1.31)	0.93 (0.59-1.46)	0.92 (0.67-1.27)

ORs and confidence intervals (CIs) were estimated using survey-weighted logistic regression. (A) Overall OR estimates in all six LGAs combined. (B) Contribution of each LGA to the combined OR estimates. [1]Model was unadjusted for any covariates, [2]Model was adjusted for age group, and [3]Model included an interaction between age group and sex to allow age to modify the effect of sex on crude coverage. ORs in bold show statistically significant difference. The odds of an outcome is the ratio of the probability the outcome happens to the probability the outcome does not happen. An odds ratio compares the relative odds of an outcome between two groups. In our case, our outcome is vaccine coverage, so the odds ratios compare the odds of vaccine coverage between two groups. Specifically, we report odds ratios of vaccine coverage comparing males to females. We interpret these odds ratios as follows: OR = 1 means no difference in the odds of vaccine coverage between males and females; OR > 1 means males have a higher odds of vaccine coverage than females; OR < 1 means males have a lower odds of vaccine coverage than females.

Ngwa MC, *et al. BMJ Global Health* 2020; 5:e002431. doi: 10.1136/bmjgh-2020-002431
