# Supplementary material for: The reactive vaccination campaign against cholera emergency in camps for internally displaced persons, Borno, Nigeria, 2017: a two-stage cluster survey
Source: BMJ Glob Health. 2020 Jun 29;5(6):e002431. doi: 10.1136/bmjgh-2020-002431 (PMC7326259; doi:10.1136/bmjgh-2020-002431)
Supplement: Supplementary data [file bmjgh-2020-002431supp009.pdf]

S5 Table: AEFI status, AEFI symptoms, symptom start, and actions taken.

Measure	Maiduguri/% (95%CI)	Jere/% (95%CI)	Konduga/% (95%CI)	Mafa/% (95%CI)	*Dikwa/% (95%CI)	Monguno/% (95%CI)	All LGAs/% (95%CI)
A. AEFI status							
Yes R1	1	2	0.4	3	0.1	1	1.2
	(0.3-1)	(0.8-4)	(0.2-0.9)	(1.2-5)	(0.0-0.4)	(0.5-2)	(0.8-2)
Yes R2	0.2	2	0.2	1	0.1	1	1.1
	(0.1-1)	(1-4)	(0.1-0.5)	(0.4-3)	(0.0-0.4)	(0.6-3)	(0.6-2)
B. AEFI symptoms							
Fever R1	59	48	60	59	-	45	50
	(28-85)	(23-72)	(25-89)	(42-75)	-	(20-73)	(33-64)
Fever R2	34	41	50	53	-	16	34
	(8-71)	(26-59)	(21-79)	(8-94)	-	(4-34)	(24-46)
Headache R1	1	18	10	28	-	8	17
	(0.1-8)	(7-35)	(0.3-44)	(15-35)	-	(1-25)	(10-26)
Headache R2	0	28	20	25	-	16	22
	0	(14-47)	(2-57)	(7-45)	-	(7-31)	(12-35)
Diarrhea R1	27	22	1	3	-	16	17
	(4-69)	(7-47)	(0.1-8)	(0.2-11)	-	(0.7-58)	(8-31)
Diarrhea R2	46	26	15	9	-	13	21
	(10-86)	(10-48)	(2-44)	(1-29)	-	(0.2-56)	(9-38)
Abdominal pain R1	38	11	49	11	-	5	13
	(6-81)	(2-33)	(19-80)	(1-36)	-	(0.1-24)	(6-24)
Abdominal pain R2	38	11	49	11	-	5	7
	(6-88)	(2-33)	(19-80)	(2-36)	-	(0.1-24)	(3-13)
Vomiting R1	15	10	31	12	-	18	13
	(3-39)	(3-25)	(8-62)	(3-27)	-	(4-43)	(7-21)
Vomiting R2	17	12	14	1	-	14	12
	(2-52)	(4-26)	(0.3-57)	(0.0-3)	-	(5-28)	(7-20)
Nausea R1	11	8	9	1	-	23	10
	(1-37)	(1-23)	(1-29)	(0.1-7)	-	(5-56)	(5-19)
Nausea R2	12	15	22	2	-	25	17
	(0.6-45)	(5-73)	(5-53)	(0.1-7)	-	(4-63)	(8-29)
C. Symptom onset/start							
Immediately R1	12	10	7	8	-	20	12
	(2-33)	(3-25)	(0.7-26)	(3-17)	-	(5-47)	(6-19)
Immediately R2	18	12	0	0.5	-	30	17
	(0.6-66)	(2-33)	0	(0.0-3)	-	(5-68)	(7-32)
< 30 mins R1	5	11	3	57	-	19	20
	(0.4-17)	(3-28)	(0.1-20)	(19-86)	-	(6-41)	(9-36)
< 30 mins R2	8	18	9	33	-	3	14
	(2-23)	(8-43)	(0.2-40)	(7-71)	-	(0.1-16)	(6-26)
After 1 hour R1	5	11	3	54	-	19	15
	(0.4-17)	(3-28)	(0.1-18)	(18-87)	-	(5-74)	(5-30)
After 1 hour R2	8	18	9	33	-	3	15
	(2-23)	(8-34)	(0.2-34)	(7-71)	-	(0.1-16)	(10-23)
After 12 hour R1	5	11	3	54	-	19	28

Ngwa MC, *et al. BMJ Global Health* 2020; 5:e002431. doi: 10.1136/bmjgh-2020-002431

	(0.4-17)	(3-28)	(0.1-20)	(19-86)	-	(6-41)	(18-40)
After 12 hour R2	8	18	9	33	-	3	28
	(2-23)	(8-34)	(0.2-40)	(7-71)	-	(0.1-16)	(15-45)
1 and 2 days R1	17	14	27	13	-	9	13
	(4-41)	(4-31)	(3-71)	(0.2-57)	-	(0.1-43)	(6-24)
1 and 2 days R2	37	15	26	25	-	13	17
	(11-70)	(4-35)	(5-64)	(3-67)	-	(1-44)	(9-29)
>3 days R1	9	9	15	1	-	23	10
	(1-33)	(4-17)	(0.6-55)	(0.1-5)	-	(7-49)	(5-17)
>3 days R2	0	7	6	3	-	3	5
	0	(0.8-24)	(0.1-40)	(0.1-18)	-	(0.1-16)	(1-12)
D. Symptom actions							
Did nothing R1	23	16	19	11	-	59	24
	(7-49)	(7-29)	(5-45)	(1-36)	-	(26-86)	(14-37)
Did nothing R2	52	20	9	8	-	50	30
	(8-94)	(10-35)	(0.2-40)	(1-25)	-	(27-73)	(18-44)
Health facility R1	29	20	6	26	-	11	19
	(4-71)	(5-71)	(0.1-32)	(6-57)	-	(0.5-43)	(1-32)
Health facility R1	14	26	53	26	-	42	31
	(2-44)	(6-58)	(16-87)	(3-67)	-	(17-70)	(18-48)
Self-med home R1	0	21	20	21	-	16	18
	0	(6-45)	(0.1-74)	(10-37)	-	(4-40)	(10-29)
Self-med home R2	0	20	13	21	-	17	18
	0	(5-46)	(0.1-63)	(6-47)	-	(7-32)	(9-30)
Saw doctor R1	24	15	4	20	-	6	14
	(2-68)	(2-41)	(0.1-21)	(8-38)	-	(0.1-30)	(6-26)
Saw doctor R2	24	7	15	3	-	0	5
	(1-77)	(4-12)	(1-48)	(0.1-18)	-	0	(2-11)
Self-med phcy R1	0	0.2	0.2	0	-	0	14
	0	(0.1-0.5)	(0-0.5)	0	-	0	(5-30)
Self-med phcy R2	0.1	0.2	0.2	0	-	0	11
	(0-0.4)	(0-0.5)	(0-0.6)	0	-	0	(3-26)
Saw CHW R1	21	2	19	22	-	0	7
	(1-68)	(0.1-9)	(0.3-73)	(7-43)	-	0	(2-17)
Saw CHW R2	0	6	0	37	-	0	7
	0	(2-14)	0	(3-90)	-	0	(2-17)
Traditional healer R1	0	2	16	0	-	0	2
	0	(0.1-8)	(1-55)	0	-	0	(0.2-5)
Traditional healer R2	6	0	0	0	-	0	0.1
	(0.1-33)	0	0	0	-	0	(0.1-2)

**There were minimal AEFI reports from Dikwa. (R1, R2) rounds 1 and 2, (phcy) pharmacy, (CHW) community health worker.*
